# 25-Hydroxycholesterol Mediates Cholesterol Metabolism to Restrict Porcine Deltacoronavirus Infection via Suppression of Transforming Growth Factor β1

**DOI:** 10.1128/spectrum.02198-22

**Published:** 2022-10-31

**Authors:** Jialu Zhang, Guanghui Yang, Xuefei Wang, Yaohong Zhu, Jiufeng Wang

**Affiliations:** a Department of Veterinary Medicine, China Agricultural University, Beijing, China; University of Florida

**Keywords:** 25-hydroxycholesterol, cholesterol metabolism, porcine deltacoronavirus, TGF-β1, lipid droplets

## Abstract

Porcine deltacoronavirus (PDCoV), an emerging enteropathogenic coronavirus in pigs, is one of the major pathogens for lethal watery diarrhea in piglets and poses a threat to public health because of its potential for interspecies transmission to humans. 25-Hydroxycholesterol (25HC), a derivative of cholesterol, exhibits multiple potential modulating host responses to pathogens, including viruses and bacteria, as well as pathogen-induced inflammation, while its antiviral effect on PDCoV and how it mediates the biological process of host cells to counter against infections remain poorly understood. Here, we thoroughly explored the antiviral effect of 25HC on PDCoV infection and tried to elucidate the underlying mechanisms. 25HC showed no toxic effect in LLC-PK1 cells and exerted antiviral ability against PDCoV infection *in vitro*. The viral cycle and time-of-addition analyses showed that 25HC mainly restricted the early and middle periods of the PDCoV postentry stage to inhibit infection. 25HC regulated disordered cholesterol metabolism induced by PDCoV infection and stimulated interferon-related lipid droplet accumulation. Transforming growth factor β1 (TGF-β1), screened by bioinformatic analyses, seemed to play an important role in PDCoV infection and was downregulated by 25HC. One interesting finding is that inhibition of TGF-β1 with the inhibitor asiaticoside exhibited a similar antiviral capacity to 25HC and demonstrated regulation of cholesterol metabolism. Taking all of the findings together, we verified the antiviral effect of 25HC on PDCoV through interference with cholesterol metabolism, which may be related to its suppression of TGFβ1.

**IMPORTANCE** As an emerging enteropathogenic coronavirus in pigs, porcine deltacoronavirus (PDCoV) causes giant economic loss in the pig industry because of lethal diarrhea and possesses the potential for transmission from animals to humans. Several pieces of evidence have suggested the antiviral potential of cholesterol-25-hydroxylase and importance of cholesterol in viral infection. This study reports that 25-hydroxycholesterol (25HC) significantly restricted PDCoV infection through modulation of cholesterol metabolism, and we identified that lipid droplets play important roles in interferon response against virus infection. Moreover, this study identified the importance of TGF-β1 in CoV infection by bioinformatic analysis and verified that the inhibition of TGF-β1 showed anti-PDCoV capacity. Moreover, we uncovered the relationship between TGF-β and cholesterol metabolism initially. Given that the importance of cholesterol in viral infection, 25HC has a great potential to treat PDCoV infection and TGF-β1 can be a crucial antiviral target.

## INTRODUCTION

Coronavirus (CoV), a sort of enveloped virus containing positive single-strand genomic RNA, has caused multiple serious pandemics in humans and animals (1), such as severe acute respiratory syndrome coronavirus 2 (SARS-CoV-2), the causative agent of COVID-19, which has been spreading from December 2019 (2). Porcine deltacoronavirus (PDCoV), an enteropathogenic virus belonging to the genus *Deltacoronavirus*, mainly infects piglets, leads to giant economic loss to the hog industry, and also threatens human health (3, 4). PDCoV has the smallest genomic RNA in coronaviruses (5), which means increased prevalence and pathogenicity. Investigation of the crystal structure of PDCoV and its receptors provided further insights into the transmission of PDCoV among species (6). Recently, infections of PDCoV among Haitian children were identified by researchers (7), suggesting the potential of PDCoV to transmit from animals to humans. In addition, several pieces of evidence have shown that PDCoV can infect both respiratory and intestinal epithelia (8), indicating that PDCoV can be an ideal model to investigate the pathogenic mechanisms and identify therapeutic targets for existing or emerging human coronaviruses, such as SARS-CoV-2.

Cholesterol is absolutely a key molecule of biological processes and cellular components, being a crucial component of plasma membranes in all animal cells, regulating membrane fluidity and interacting with adjacent lipids (9, 10). Cholesterol metabolism plays important roles in both innate and adaptive immune responses (11, 12). Because of its popularity in biological activity, an abnormal index of cholesterol has been connected with multiple diseases; for instance, hypercholesterolemia implies an increased risk of inflammatory bowel disease and cancer (13). Meanwhile, many viruses utilize membrane cholesterol to enter the host and transmit virus particles between organelles to accomplish the infection (14–17). However, the underlying mechanism regarding its utilization and concrete functions of cholesterol in the context of virus infection is still elusive.

Recently, oxysterols have received increasing attention among cholesterol metabolites. As cholesterol oxidation products, previous studies have mainly focused on their physiological process and their correlations with nutritional and metabolic diseases (18). Nowadays, except for their proapoptotic and proinflammatory effects, more aspects of their beneficial function have been uncovered (19). Side-chain cholesterol oxides, such as 24-, 25-, and 27-hydroxycholesterol (24HC, 25HC, and 27HC, respectively), exhibited noteworthy antibacterial and antiviral potential. COVID-19 patients showed significantly reduced 27HC content in blood compared to the control group, whereas 27HC complexed with 2-hydroxypropyl-β-cyclodextrin indicated antiviral capacity against SARS-CoV-2 in Vero E6 cells (20). 25HC can provide innate immunity against Listeria monocytogenes infection by mobilizing accessible cholesterol on cell membrane (21). Furthermore, 25HC showed effective antiviral activity against several viruses, including hepatitis C virus (HCV) (22), Andes virus (14), influenza virus (23), vesicular stomatitis virus (VSV) (24), and SARS-CoV-2 (25). Besides, cholesterol-25-hydroxylase (CH25H), an enzyme that synthesizes the 25HC (26), also possesses significant antiviral activity against porcine viruses, such as porcine epidemic diarrheal virus (PEDV) (27), porcine reproductive and respiratory syndrome virus (PRRSV) (28), and PDCoV (29). Therefore, we speculated that 25HC has an inhibitory effect on PDCoV replication and are curious about the corresponding mechanism.

In this study, we explored the antiviral properties of 25HC on PDCoV infection and found that it mainly took effect in the postentry stage of PDCoV life cycle. In addition, with prolonged treatment 25HC displayed better antiviral ability. We demonstrated that 25HC could restore the homeostasis of disordered cholesterol metabolism induced by PDCoV, which might be mediated by transforming growth factor β1 (TGF-β1). Our findings provide new insights into the mechanisms of viral infection and the development of oxysterols as antiviral agents.

## RESULTS

### 25HC inhibits PDCoV infection in LLC-PK1 cells.

**FIG 1:**
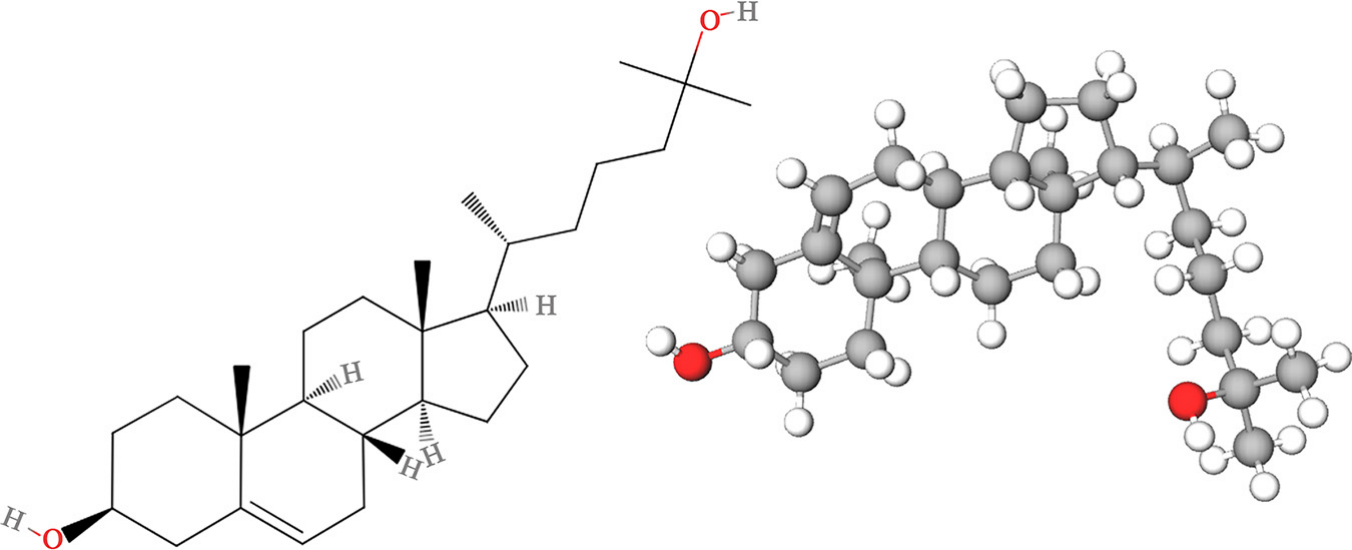
Chemical structure of 25-hydroxycholesterol shown as 2D and 3D models.

**FIG 2:**
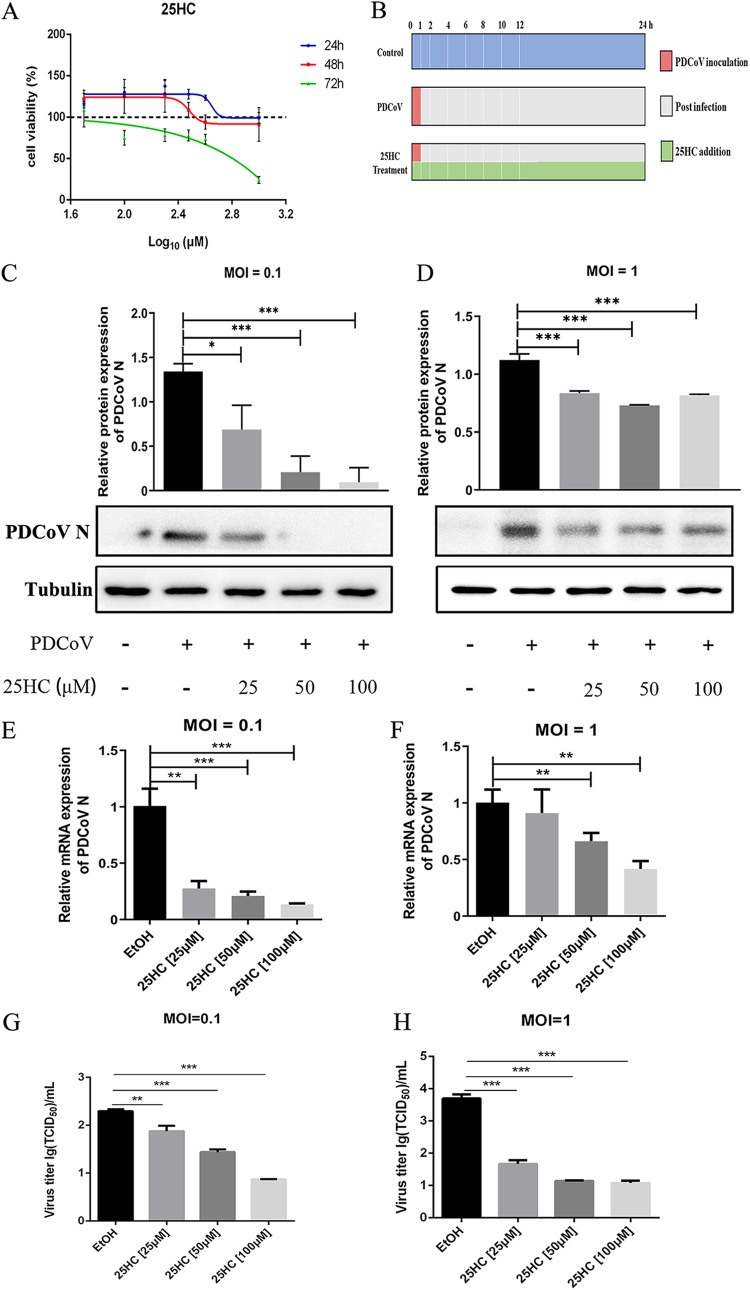
25HC inhibits PDCoV infection in LLC-PK1 cells. (A) LLC-PK1 cells were treated with 25HC (50 to 1,000 μM) for 24 to 72 h. Cell viability was measured by the CCK-8 assay. (B) Schematic diagram of PDCoV infection and 25HC addition. (C and D) LLC-PK1 cells were infected with PDCoV at an MOI of 0.1 or 1 and treated with 25HC (25 to 100 μM) following the timeline in panel B. PDCoV N and tubulin were assessed by Western blotting, and the immunoblotting bands were quantified by ImageJ. (E and F) Cells were treated the same as in panels C and D. The transcription level of PDCoV N was measured by qRT-PCR analysis. EtOH represents the virus control group. (G and H) Cells were treated the same as in panels C and D. Virus titers were assessed by TCID_50_ assay. Data represent means ± SD from three independent experiments. *, *P* < 0.05; **, *P* < 0.01; ***, *P* < 0.001.

The chemical structure of 25HC is shown in Fig. 1. We first evaluated the effects of 25HC on cell viability. LLC-PK1 cells were treated with 25HC (50, 100, 200, 300, 400, and 1,000 μM) for 24 to 72 h, and cytotoxicity was evaluated in three independent experiments by the Cell Counting Kit-8 (CCK-8) assay. Treatment with various concentrations of 25HC for 24 h and 48 h exhibited no significant cytotoxicity to LLC-PK1 cells, whereas treatment with 25HC at concentrations from 100 to 1,000 μM for 72 h showed cytotoxicity (Fig. 2A). To determine the inhibitory effect of 25HC on PDCoV infection, nontoxic concentrations of 25HC (25, 50, and 100 μM) were added along with PDCoV at different multiplicities of infection (MOI) for 24 h (Fig. 2B). The protein and mRNA levels of PDCoV N were analyzed by Western blotting and quantitative reverse transcription-PCR (qRT-PCR), respectively. The Western blot analysis showed that 25HC effectively reduced expression of PDCoV N protein at different MOI in a dose-dependent manner (Fig. 2C and D). The qRT-PCR analysis indicated that the transcript of PDCoV N was dramatically downregulated in 25HC treatment groups, and 25HC at 50 and 100 μM displayed better inhibitory activities (Fig. 2E and F). Moreover, we verified the antiviral capacity of 25HC by the virus titration assay and found that 25HC resulted in the decline in the progeny virus production of PDCoV (Fig. 2G and H). The results presented above suggested that 25HC possessed antiviral ability against PDCoV. Taking the effectiveness into account, we chose 50 μM 25HC to conduct the following experiments.

### 25HC inhibits PDCoV replication and release at the postentry stage.

**FIG 3:**
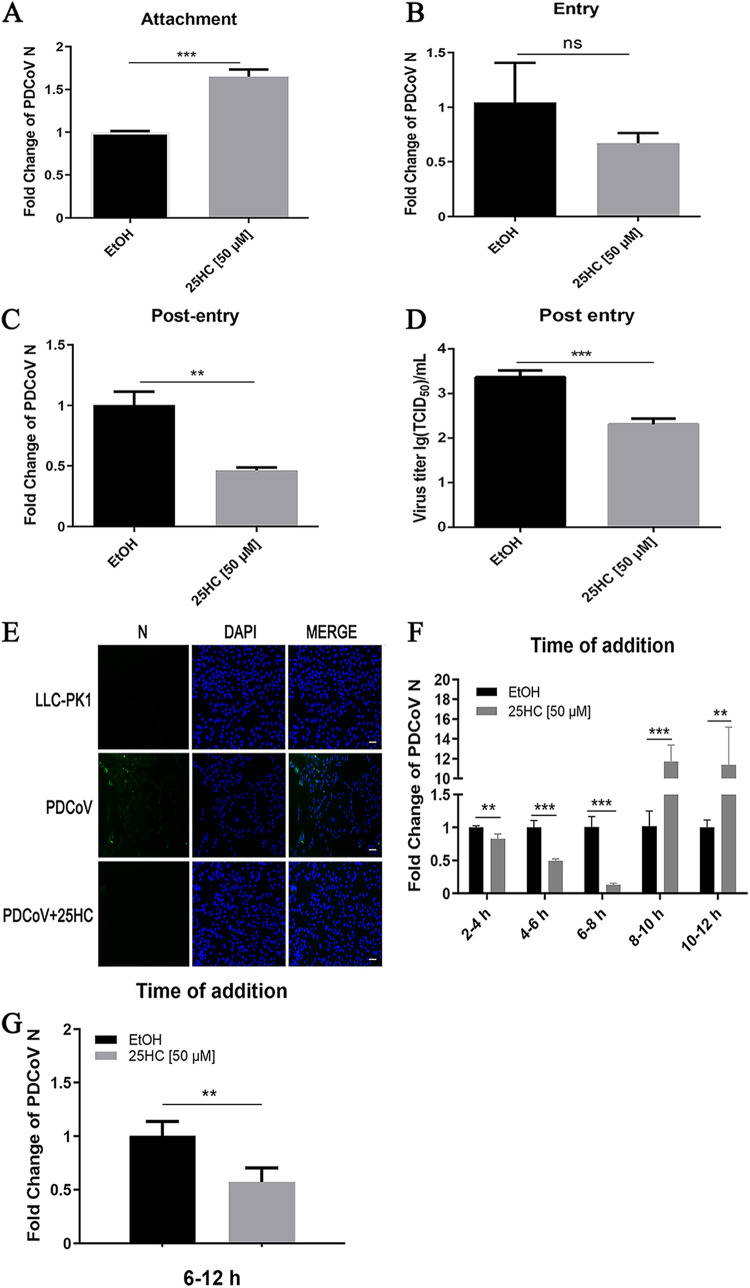
25HC inhibits PDCoV replication and release at the postentry stage. (A to C) The relative levels of viral RNA expression in LLC-PK1 cells treated with 50 μM 25HC during viral cycle, including attachment (A), entry (B), and postentry (C) stages, were assessed by qRT-PCR with primers targeting the PDCoV N gene and GAPDH. (D) The virus titer in cell supernatants treated with 25HC in viral postentry stage was determined by TCID_50_ assay. (E) The inhibitory effect of 25HC on PDCoV replication during the postentry stage was assessed by immunofluorescence staining. Scale bar, 50 μm. (F and G) The inhibitory effect of 25HC on PDCoV infection was determined by time-of-addition analysis from 2 to 12 hpi during the postentry stage. Data represent means ± SD from three independent experiments. **, *P* < 0.01; ***, *P* < 0.001; ns, not significant.

Next, we investigated the stage of the PDCoV life cycle targeted by 25HC. In general, the viral cycle was divided into three stages, including attachment (at 4°C), entry (the first hour at 37°C after virus inoculation), and postentry (1 to 12 h postinfection [hpi]). qRT-PCR was applied to analyze the inhibitory effect of 25HC on PDCoV attachment, entry, and postentry stages. During the attachment stage, 25HC showed no antiviral activity and the transcript of PDCoV N in 25HC-treated group even significantly increased compared with that in the ethanol (EtOH) group (virus control group) (Fig. 3A), whereas, treatment with 25HC at the postentry stage significantly reduced PDCoV N mRNA levels (Fig. 3B) and viral titers in cell supernatants (Fig. 3D), indicating that 25HC can interfere with PDCoV replication and progeny release. These results were further confirmed by immunofluorescent antibody (IFA) analysis that 25HC treatment significantly reduced infected cells (Fig. 3E). Furthermore, to investigate at which period can 25HC exert its antiviral effect in the postentry stage of PDCoV, we performed time-of-addition experiments at 2 to 12 hpi in LLC-PK1 cells. The mRNA levels of PDCoV, which indicate the level of virus content, were significantly reduced when infected cells were treated with 25HC during 2 to 4, 4 to 6, and 6 to 8 hpi, whereas 25HC exerted no antiviral activities during 8 to 10 and 10 to 12 hpi (Fig. 3F). In order to figure out whether prolonged 25HC treatment has an antiviral ability, we performed another time-of-addition experiment at 6 to 12 hpi. Of interest, with prolonged 25HC addition PDCoV N mRNA significantly declined during 6 to 12 hpi. Taken together, 25HC mainly exerts its antiviral effect at the early and middle parts of the PDCoV postentry stage, thwarting PDCoV replication and progeny release.

### 25HC rescues PDCoV-induced cholesterol metabolism disorder.

**FIG 4:**
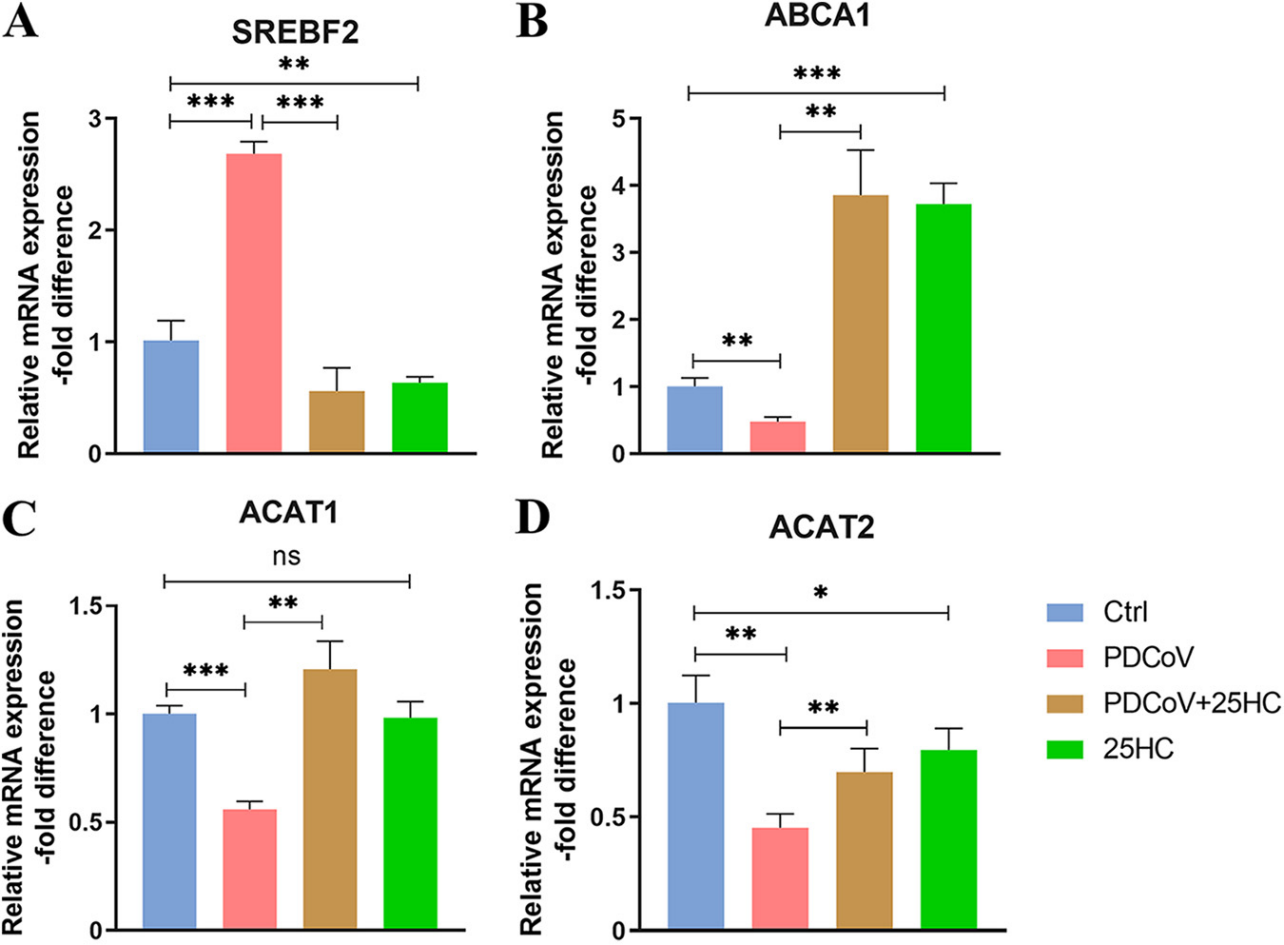
25HC rescues PDCoV-induced cholesterol metabolism disorder. (A) Effect of 25HC on mRNA expression of the gene coding for SREBF2, essential for cholesterol synthesis, was assessed by qRT-PCR. (B) Effect of 25HC on mRNA expression of the gene coding for ABCA1, essential for cholesterol efflux, was assessed by qRT-PCR. (C and D) Effects of 25HC on mRNA expression of the genes coding for ACAT1 and ACAT2, essential for cholesterol esterification, were assessed by qRT-PCR. Data represent means ± SD from three independent experiments. **, *P* < 0.01; ***, *P* < 0.001.

Given the importance of cholesterol to viral infection and the fact that 25HC is a metabolite of cholesterol, qRT-PCR analysis was applied to measure the mRNA levels of the indicated genes of cholesterol metabolism, including sterol regulatory element binding transcription factor 2 (SREBF2) for synthesis, ATP binding cassette subfamily A member 1 (ABCA1) for efflux, and acetyl-CoA acetyltransferase 1 (ACAT1), and acetyl-CoA acetyltransferase 2 (ACAT2) for esterification. Compared with the control group, the mRNA expression level of SREBF2 was upregulated, while the mRNA expression levels of ABCA1, ACAT1, and ACAT2 were downregulated after PDCoV inoculation at 37°C for 24 h (Fig. 4). Of note, the application of 25HC along with PDCoV inoculation significantly reduced the gene expression of SREBF2 and significantly increased the gene expressions of ABCA1, ACAT1, and ACAT2 compared with the PDCoV group, in addition, the individual administration of 25HC significantly reduced the expression of SREBF2 and ACAT2 and significantly upregulated the expression of ABCA1 (Fig. 4). These results suggested that 25HC can modulate the disturbance of cholesterol metabolism caused by PDCoV infection.

### 25HC induces LD accumulation accompanied by elevated interferon mRNA during PDCoV infection.

**FIG 5:**
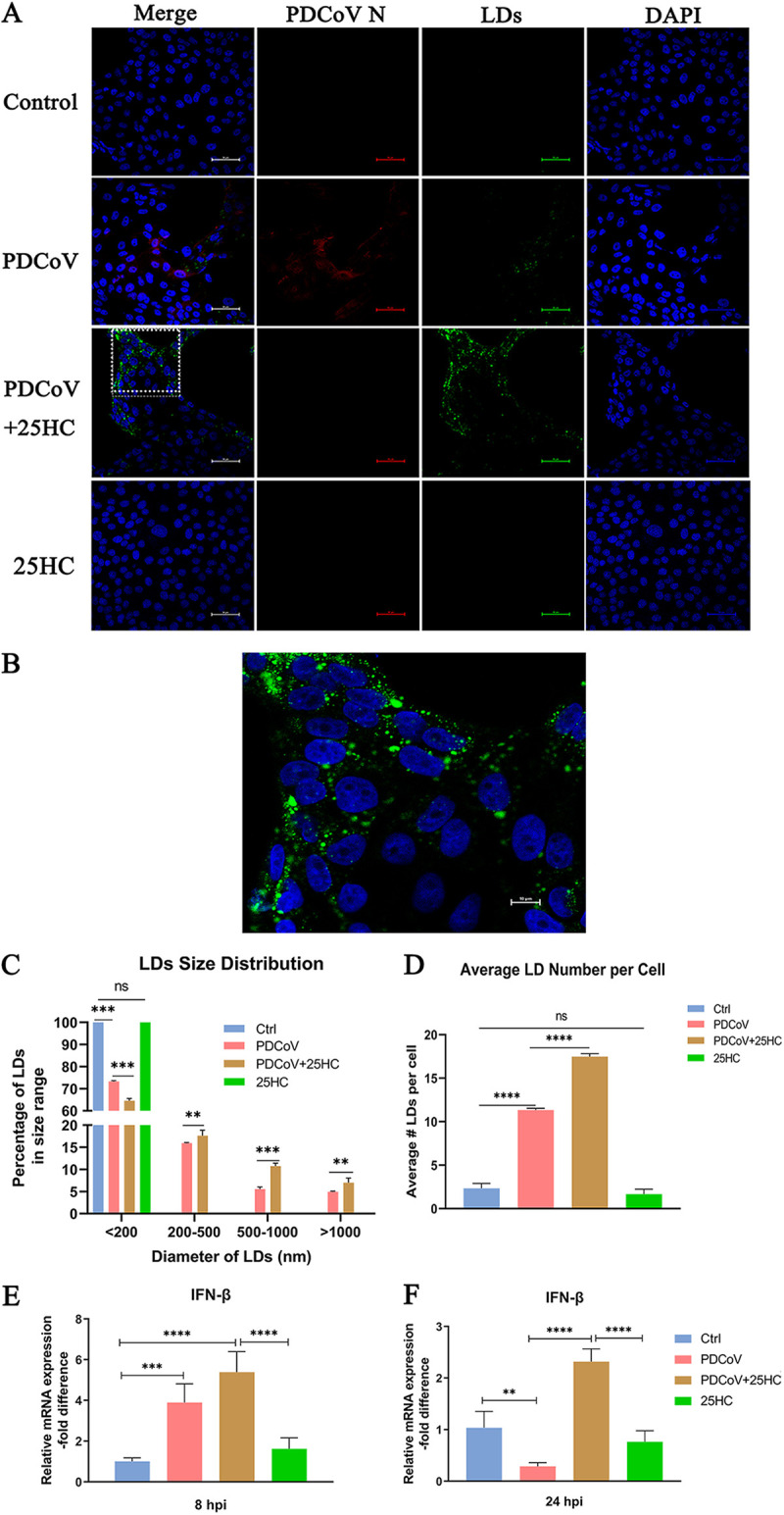
25HC induces LD accumulation accompanied by elevated interferon mRNA during PDCoV infection. (A) LLC-PK1 cells were infected with PDCoV and treated with 25HC for 8 h. Lipid droplets (LDs) were stained with BODIPY 493/503, and cell nuclei were stained with DAPI. Scale bar, 50 μm. (B) Enlargement of the boxed region in panel A. Scale bar, 10 μm. (C and D) LD size distribution (C) and average LD number per cell (D) were analyzed using ImageJ software from three replicates. (E) Cells were treated as in panel A, and qRT-PCR was utilized to quantify the mRNA expressions of IFN-β at 8 hpi. (F) Cells were incubated with PDCoV and treated with 25HC for 24 h; the mRNA expression of IFN-β was measured by qRT-PCR analysis. Data represent means ± SD from three independent experiments. **, *P* < 0.01; ***, *P* < 0.001.

Lipid droplets (LDs) showed a connection with the early response of cells to viral infection (30), while their roles in PDCoV infection remain unclear. Herein, we utilized BODIPY 493/503 to quantify the numbers and size distribution of LDs at 8 hpi to characterize the LD accumulation following PDCoV infection. LDs were upregulated following PDCoV infection. Moreover, the number of LDs was notably increased in the 25HC treatment group compared with the PDCoV group (Fig. 5A and B). The normal LLC-PK1 cells had a low basal level of LDs and were mainly distributed in the size range of <200 nm in diameter, whereas the PDCoV group and 25HC treatment group had plenty of LDs with diameters ranging from <200 to >1,000 nm, and the individual addition of 25HC did not change the LD level of the cells (Fig. 5C). The percentage of LDs with diameters of <200 nm in the PDCoV group significantly exceeded that in the 25HC-treated group. However, it was noteworthy that 25HC significantly increased the percentages of LDs with diameters ranging from 200 to more than >1,000 nm compared with the PDCoV infection group (Fig. 5C). Subsequently, we calculated the average number of LDs per cell, and the results showed that 25HC induced a substantial accumulation of LDs in PDCoV-infected cells, which was significantly different from the PDCoV group (Fig. 5D).

Next, to determine if the increased LDs following PDCoV infection and 25HC administration play antiviral roles in cells, we tested the mRNA levels of interferon beta (IFN-β). Interestingly, IFN-β transcription increased significantly at 8 hpi, which was consistent with LD upregulation (Fig. 5E). To determine whether the upregulation of IFN caused by PDCoV infection consistently exists, we measured the IFN-β mRNA expression at 24 hpi. The results showed that virus-induced IFN-β expression significantly declined at 24 hpi, while the addition of 25HC brought a continuous antiviral response with markedly enhanced IFN-β mRNA expression (Fig. 5F). Of note, individual addition of 25HC did not trigger significant changes in IFN-β mRNA compared with the control group (Fig. 5E and F). These results suggested that 25HC can stimulate enhancement of cellular LDs during PDCoV infection, which correlated with interferon accumulation.

### Bioinformatic analyses identify intersecting genes of highly pathogenic CoVs.

**FIG 6:**
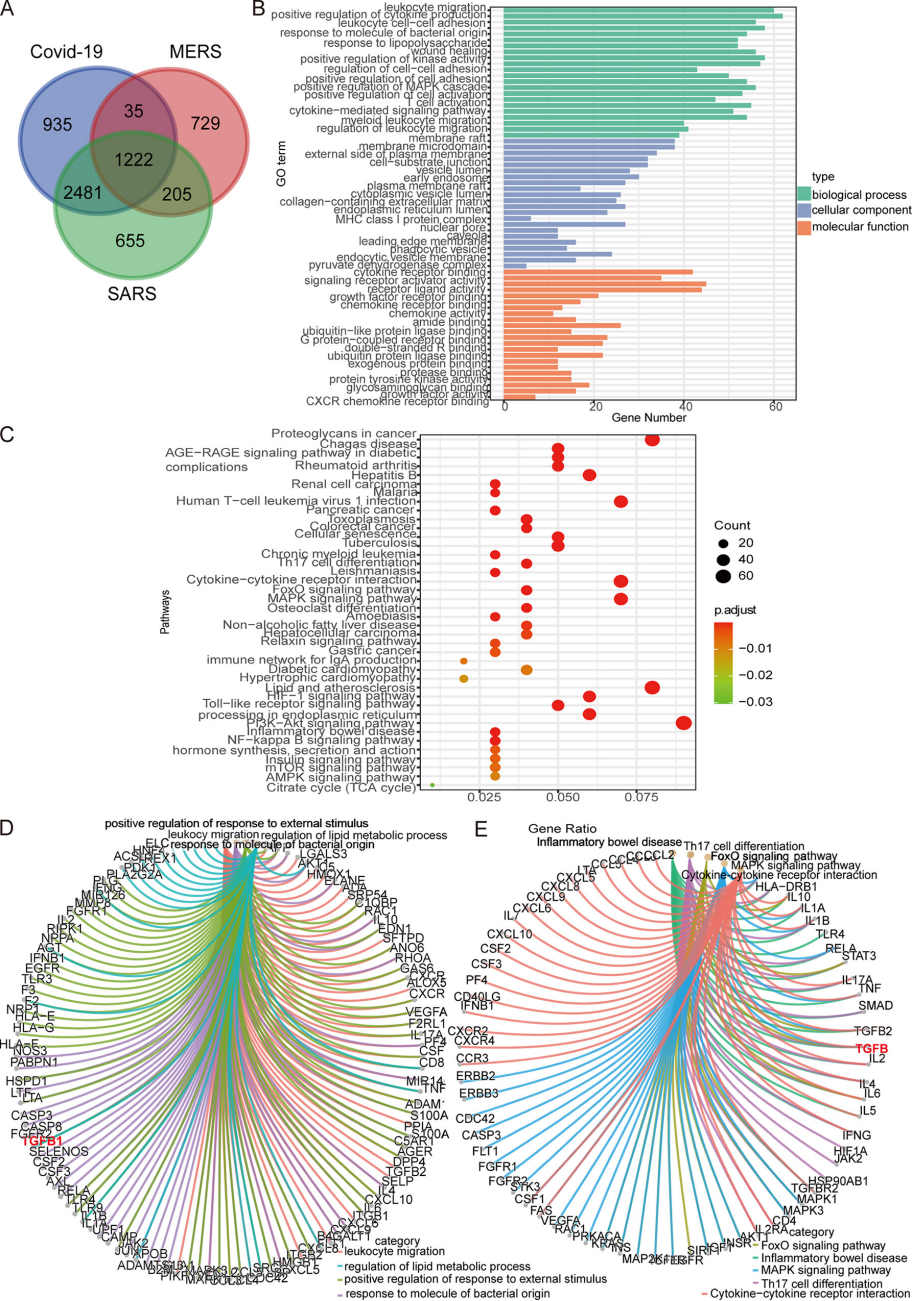
Bioinformatic analyses identify intersecting genes of highly pathogenic CoVs. (A) COVID-19-, SARS-, and MERS-related genes were collected from three databases, and a Venn diagram was made to depict overlapping genes. (B) Gene Ontology analysis of intersecting genes and part of the significantly enriched GO terms are displayed. (C) Kyoto Encyclopedia of Genes and Genomes (KEGG) pathway analysis of intersecting genes and part of the significantly enriched pathways are displayed. (D and E) cnetplots of selected GO terms and KEGG pathways with related genes.

First, we collected genes associated with COVID-19, SARS, and Middle East respiratory syndrome (MERS) from the GeneCards, OMIM, and NCBI databases, respectively. A comparison of these three gene clusters screened 1,222 intersecting genes involved in COVID-19, SARS, and MERS (Fig. 6A). GO and KEGG analyses of the 1,222 intersecting genes revealed that CoV affected a series of biological processes, molecular functions, and cellular components, including positive regulation of cytokine production, response to molecule of bacterial origin, regulation of cell-cell adhesion, the cytokine-mediated signaling pathway, membrane raft, early endosome, endocytic vesicle, cytokine activity, signaling receptor activator activity, chemokine receptor binding, and growth factor activity (Fig. 6B). Additionally, in the KEGG analysis results, we display 40 KEGG pathways (adjusted *P* value of <0.05), which we were interested in, related to the intersecting genes. These pathways included those related to proteoglycans in cancer, rheumatoid arthritis, malaria, Th17 cell differentiation, cytokine-cytokine receptor interaction, FoxO signaling pathway, the mitogen-activated protein kinase (MAPK) signaling pathway, protein processing in the endoplasmic reticulum, inflammatory bowel disease, the NF-κB signaling pathway, and growth hormone synthesis, secretion, and action (Fig. 6C). Next, we mapped networks of GO terms (leukocyte migration, positive regulation of response to external stimulus, regulation of lipid metabolic process and response to molecule of bacterial origin) and KEGG pathways (cytokine-cytokine receptor interaction, FoxO signaling pathway, inflammatory bowel disease, MAPK signaling pathway, and Th17 cell differentiation) with related genes that we were interested in. Taking both of these networks into account, we found that transforming growth factor β1 (TGF-β1) was iteratively enriched in both GO terms and KEGG pathways (Fig. 6D and E).

### 25HC negatively regulates PDCoV-induced TGF-β1/Smad3 pathway.

**FIG 7:**
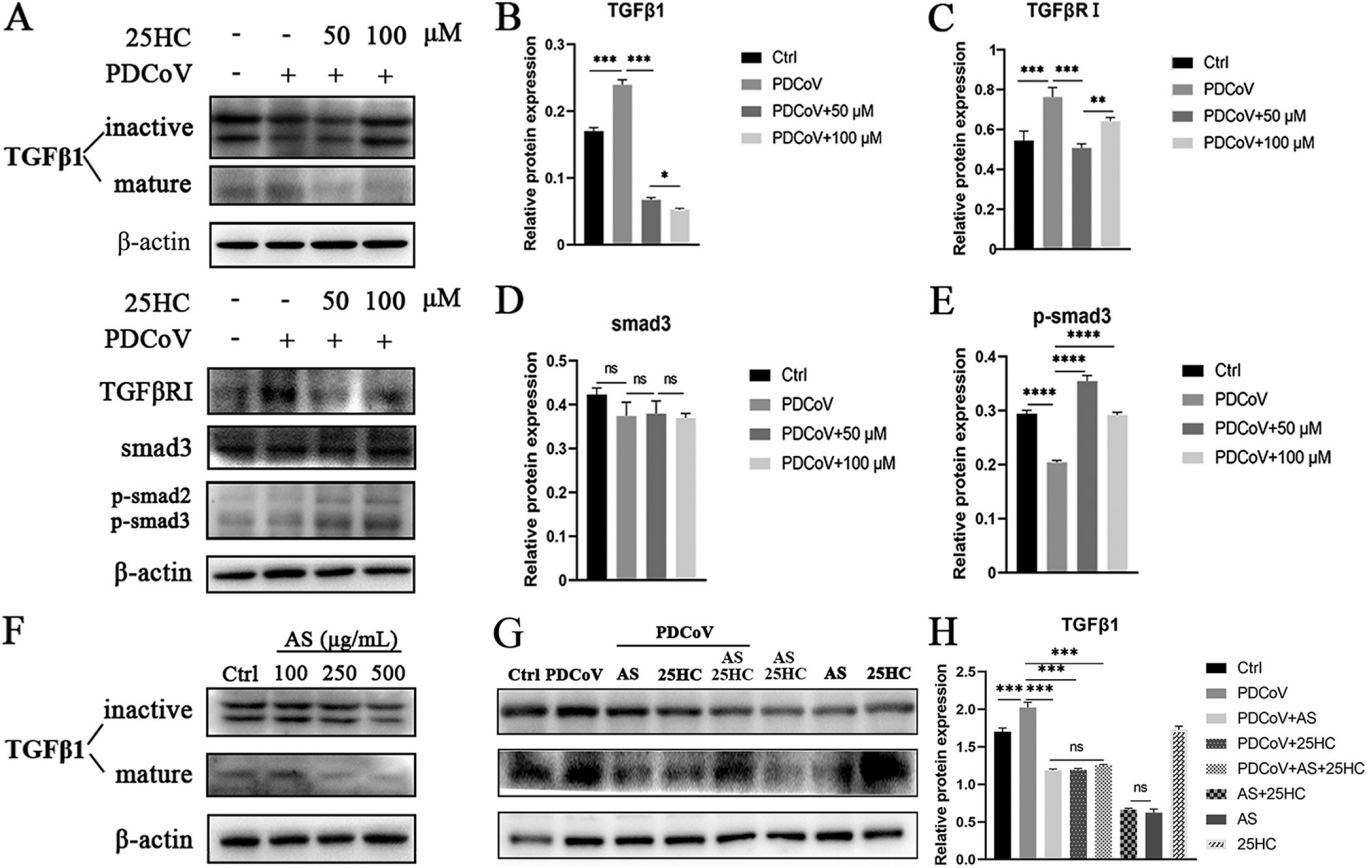
25HC inhibits PDCoV-induced TGF-β1/Smad3 pathway activation. (A) LLC-PK1 cells were infected with PDCoV and treated with different concentrations of 25HC for 24 h. The levels of protein expression of mature TGF-β1, TGF-βRI, Smad3, and p-Smad3 were determined by Western blotting analyses. (B to E) Quantification of immunoblotting bands in panel A. (F) LLC-PK1 cells were treated with different concentrations of AS for 24 h to determine its inhibitory effect on TGF-β1. The protein expression of TGF-β1 was assessed by Western blotting. (G and H) Cells were infected with PDCoV and treated with 25HC or AS for 24 h, and Western blotting was utilized to assess expression of TGF-β1 (G); the quantification is displayed in panel H. Data represent means ± SD from three independent experiments, *, *P* < 0.05; **, *P* < 0.01; ***, *P* < 0.001; ****, *P* < 0.0001; ns, not significant.

According to the results obtained from bioinformatic analyses, TGF-β1 might play important roles in CoV infection. We attempted to determine whether there was a correlation between TGF-β1 and PDCoV infection and the therapeutic effect of 25HC. Western blotting was applied to measure the protein level of TGF-β1 after PDCoV infection and 25HC treatment at 24 hpi. The protein expression of mature TGF-β1 was upregulated by PDCoV infection: different concentrations of 25HC significantly eliminated the PDCoV-induced enhancement of TGF-β1 expression (Fig. 7A and B). It is known that TGF-β is linked to TGF-β receptor (TGF-βR) activation and then phosphorylates Smad proteins to regulate transcription (31). Accordingly, we assessed the protein expressions of TGF-βRI, Smad3, and phosphorylated Smad3 (p-Smad3). TGF-βRI expression displayed a similar trend to TGF-β1 in the PDCoV and 25HC treatment groups (Fig. 7A and C). Furthermore, PDCoV infection and 25HC treatment showed no effect on expression of Smad3, but affected the expression of its phosphorylation; of interest, p-Smad3 was inhibited after infection, while this situation was restored by 25HC (Fig. 7A, D, and E). These results indicated that 25HC was a negative regulator of the PDCoV-induced TGF-β1/Smad3 pathway.

### 25HC inhibits PDCoV proliferation through inhibition of TGF-β.

**FIG 8:**
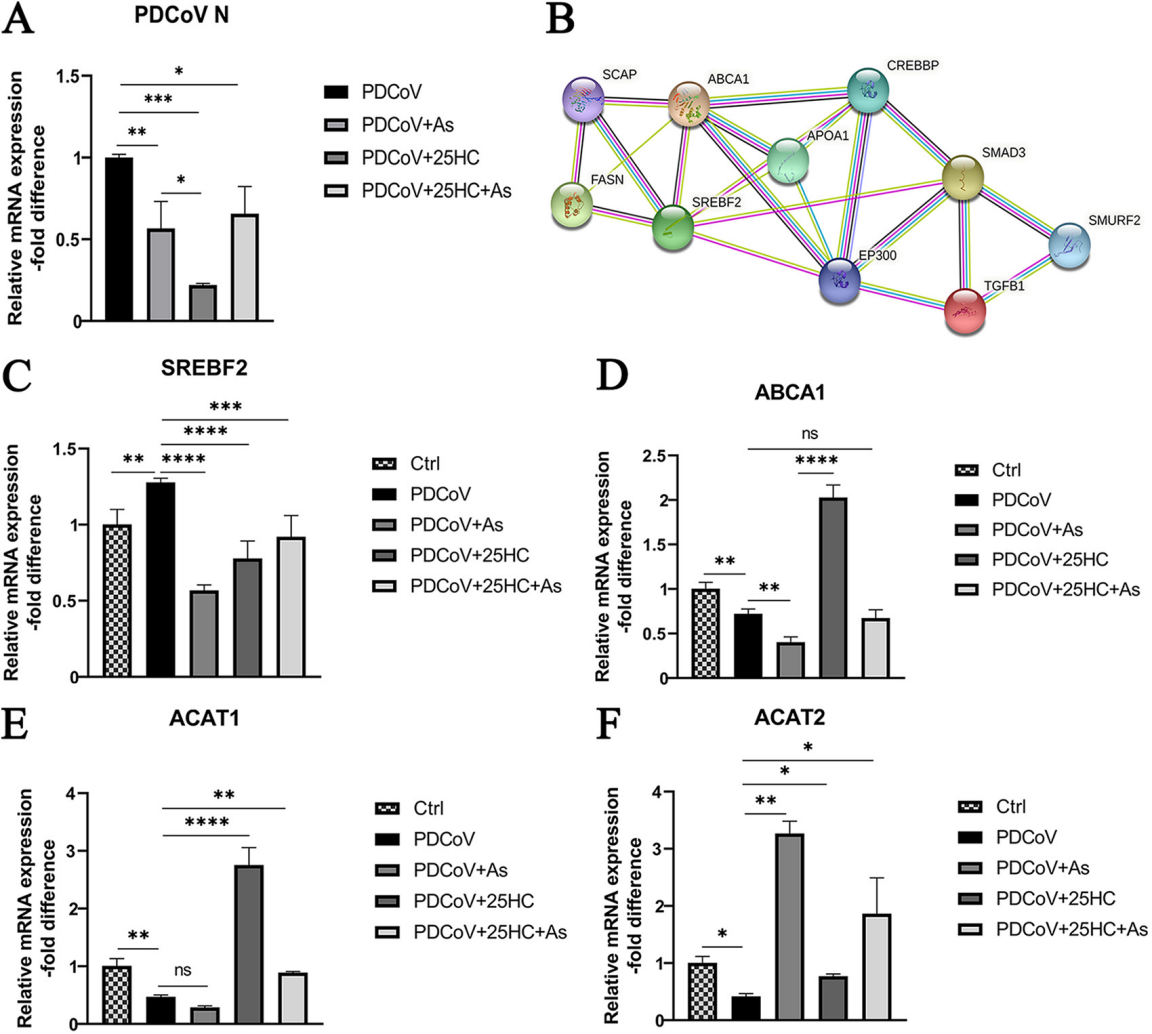
25HC inhibits PDCoV proliferation through inhibition of TGF-β1. (A) LLC-PK1 cells were infected with PDCoV and treated with 25HC and/or AS. The mRNA expression of PDCoV N was assessed by qRT-PCR analysis. (B) The correlation of TGF-β1/Smad3 with cholesterol metabolic genes was demonstrated by protein-protein interaction (PPI) analysis based on the STRING database. (C to F) Cells were treated as in panel A, and the levels of mRNA expression of genes coding for SREBF2, ABCA1, ACAT1, and ACAT2 were determined using qRT-PCR analysis. Data represent means ± SD from three independent experiments. *, *P* < 0.05; **, *P* < 0.01; ***, *P* < 0.001; ****, *P* < 0.0001.

In order to investigate whether the antiviral activity of 25HC is related to its suppression of TGF-β activation, we treated LLC-PK1 cells with the TGF-β inhibitor asiaticoside (AS) (MedChemExpress). As shown in Fig. 7F, AS significantly inhibited the expression of mature TGF-β1 at 250 and 500 μg/mL, and we applied 250 μg/mL of AS for subsequent experiments. AS application significantly reduced the expression of mature TGF-β1 after PDCoV infection, which was similar to treatment with 25HC (Fig. 7G and H). Of note, addition of both AS and 25HC to PDCoV-infected cells caused no significant difference compared with individual addition of AS or 25HC. Furthermore, without PDCoV infection, addition of both AS and 25HC induced a similar level of expression of mature TGF-β1 to single addition of AS (Fig. 7H). Meanwhile, qRT-PCR analysis showed that AS significantly reduced the transcription level of PDCoV N, while the simultaneous addition of AS and 25HC exhibited a less inhibitory effect than the individual addition of 25HC (Fig. 8A). Based on the above results, we speculated that the inhibition of PDCoV-induced TGF-β1 activation might be one of the mechanisms by which 25HC exerts its anti-PDCoV effect.

STRING analysis was applied to determine the network between TGF-β/Smad pathway and cholesterol metabolism; it indicated that TGF-β1/Smad3 affected cholesterol metabolism through regulation of SREBF2 (Fig. 8B). We further explored the role of TGF-β in cholesterol metabolism. In the presence of PDCoV, individual addition of AS or 25HC resulted in significant reduction of SREBF2 mRNA expression (Fig. 8C). AS treatment significantly downregulated the expression of ABCA1, while 25HC significantly increased its expression; these effects seemed to be counteracted when both 25HC and AS were present (Fig. 8D). Moreover, AS slightly reduced ACAT1 mRNA with no significance, while addition of both AS and 25HC caused a significant decline in the expression of ACAT1 mRNA (Fig. 8E). Interestingly, treatment with AS with or without 25HC could significantly upregulate the expression of ACAT2 (Fig. 8F). These data suggested the regulatory effect of TGF-β1 on cholesterol metabolism.

## DISCUSSION

Cholesterol plays important roles in maintaining normal biological processes and transmitting signals in cells; several pieces of evidence have demonstrated that homeostasis of cholesterol is critical to cell immunity against bacteria and viruses. Oxysterol, a form of oxidized cholesterol, has exerted considerable effects on multiple viral infection (32–34). CH25H, the enzyme regulating synthesis of 25HC, can suppress infection with PDCoV (29). However, the inhibitory effect of 25HC on PDCoV infection and the role of cholesterol in this process have not been fully elucidated. In this study, we characterized the anti-PDCoV function of 25HC and the corresponding mechanisms. 25HC exerted a prominent antiviral effect against PDCoV infection: it mainly interfered with virus replication and release in the postentry stage, but not virus attachment and entry. Considering that 25HC is a metabolite of cholesterol, we speculated that 25HC might inhibit PDCoV proliferation by regulating cellular cholesterol metabolism.

Cellular cholesterol homeostasis is maintained by the coordination of different parts of cholesterol metabolism, including cholesterol biosynthesis, uptake, efflux, and esterification (35). These parts always coregulated to supply sufficient cholesterol to cells and avoid excessive accumulation. Our findings in the present study demonstrated that PDCoV infection caused a disturbance in cholesterol metabolism, including upregulation of cholesterol synthesis and downregulation of esterification and efflux, while 25HC possessed the ability to restore cholesterol metabolism to maintain the homeostasis. Lipid droplets (LDs) have become well known in recent years for their ability to store lipid; however, nowadays they are more considered highly dynamic organelles involved in multiple functional pathways and cellular responses (36, 37). In this study, we observed LD accumulation early in PDCoV infection, and 25HC treatment further promoted the accumulation of LDs. Besides, we found that IFN-β expression was significantly elevated early in PDCoV infection and significantly decreased at 24 hpi, which was consistent with previous studies (30, 38, 39). In contrast, treatment with 25HC during PDCoV infection consistently promoted the expression of IFN-β and the effects of 25HC on LD accumulation, and interferon expression showed similar trends to some extent, which indicated the promotion effect of LD accumulation on interferon production. It is interesting that we found the individual application of 25HC did not affect the levels of LDs and IFN-β in cells; the interferon in the organisms is dynamic, and it seems that 25HC might not induce IFN-β expression in healthy organisms. Besides, interferons can mediate cholesterol reprogramming by decreasing cholesterol biosynthesis rapidly and promoting esterification of cholesterol in LDs to maintain cholesterol homeostasis against microbial infection (40–42). In the future, we will further investigate the interaction between interferon and cholesterol metabolism during PDCoV infection.

Transforming growth factor β (TGF-β) signaling controls cell behavior and development, TGF-β-related cytokines also exert vital effects on cell proliferation, morphogenesis, and tissue regeneration (31). However, the relationship between TGF-β signaling and immune response in viral infection is still not well understood. In the bioinformatic analyses, we found that TGF-β1 participated in many functional pathways in CoV infection; besides, abundant evidence has illustrated the importance of TGF-β for viral infection (43–46). Our data suggested that PDCoV-induced regulation of the TGF-β/Smad3 signaling pathway, including upregulation of TGF-β1 and TGF-βRI and downregulation of p-Smad3, was reversed by the treatment of 25HC and the inhibitory effect of 25HC on TGF-β1 was similar to that of AS. One interesting finding is that inhibition of TGF-β1 by AS exerted antiviral activity against PDCoV: the combination of 25HC and AS showed a similar inhibitory effect to individual addition of AS, but there was less of an effect than individual addition of 25HC, which indicated the inhibitory effect of 25HC was partially accomplished by reducing the expression of TGF-β1. As STRING analysis predicted, SREBF2 and ABCA1 might be the key molecules for the interaction between cholesterol metabolism and the TGF-β signaling pathway. The decreased antiviral effect of adding 25HC and AS simultaneously might be counteracted by their opposite effect on ABCA1. However, the inhibitory effect of AS on SREBF2 was similar to the effect of 25HC, demonstrating the regulatory relationship between TGF-β1 and SREBF2, which was also implied in earlier studies (47, 48): thus, 25HC might exert anti-PDCoV ability by affecting the TGF-β1/SREBF2 axis. Taking these clues together, we speculated that 25HC suppresses PDCoV infection by reprogramming cholesterol metabolism partially via inhibition of TGF-β1. However, deeper work is required in the future to uncover the role of TGF-β signaling in virus infection and its interaction with cholesterol metabolism.

In conclusion, our study provides evidence that 25HC reprograms the cholesterol metabolism to exert an anti-PDCoV effect, in which the TGF-β signaling pathway might be involved. Given the importance of TGF-β and cholesterol to cell function, we propose that signal-specific reprogramming of cellular cholesterol will generally be a critical strategy for cells to resist pathogens.

## MATERIALS AND METHODS

### Reagents.

The TransStart one-step gDNA removal and cDNA synthesis supermix kit (AT311-02) and TransStart Tip Green qPCR supermix (AQ142-21) were ordered from TransGen Biotech (Beijing, China), The total RNA kit was ordered from Omega (Guangzhou, China), BODIPY 493/503 (GC42959) was purchased from GlpBio (Montclair, CA, USA), DAPI (4′,6-diamidino-2-phenylindole) (C0060) was ordered from Solarbio Science & Technology (Beijing, China), and 25-hydroxycholesterol (25HC) (HY-113134) and asiaticoside (AS) (HY-N0439) were ordered from MedChemExpress (Monmouth Junction, NJ, USA).

### Antibodies.

Anti-PDCoV N antibody was purchased from Medgene labs (Brookings, SD, USA), anti-TGF-β1 (WL02998), anti-Smad3 (WL02288), anti-phospho-Smad3/Smad2 (WL02305), and anti-TGF-βRI (WL03150) were ordered from Wanleibio (Shenyang, China), and anti-β-actin (60008-1-AP), antitubulin (10068-1-AP), horseradish peroxidase (HRP)-conjugated goat anti-mouse IgG (SA00001-1), and anti-rabbit IgG (SA00001-2) were ordered from Proteintech (Wuhan, China). Anti-mouse IgG antibody labeled with Alexa Fluor 555 (A21424) and Alexa Fluor 488 (A21429) and anti-rabbit IgG labeled with Alexa Fluor 488 (A11034) were ordered from Thermo Fisher Scientific. The aforementioned antibodies were used at dilutions of 1:300 for immunofluorescence staining and 1:1,500 for immunoblotting analysis.

### Cell culture and virus infection.

LLC-PK1 cells (ATCC CL-101) from ATCC (the American Type Culture Collection) was cultured in Dulbecco’s modified Eagle’s medium (DMEM) (Gibco, Gaithersburg, MD, USA) supplemented with 1% antibiotic-antimycotic (Gibco, USA), 1% nonessential amino acids solution (NEAA) (Gibco, USA), and 10% fetal bovine serum (FBS) (Gibco, Fitzroy North, Victoria, Australia). The PDCoV CHN-HN-1601 strain (GenBank accession no. MG832584) was provided by Hanchun Yang, China Agricultural University, Beijing, China. The PDCoV strain was used to infect LLC-PK1 cells at an MOI of 0.1 or 1. After incubation for 1 h at 37°C in a 5% CO_2_ incubator, cells were washed three times to remove the unbound viruses and cultured in maintenance medium (DMEM supplemented with 0.4% trypsin) at 37°C.

### Cell viability analysis.

Cell viability was evaluated with CCK-8 assay (Beyotime, Beijing, China). The cells were seeded at 1 × 10^4^ per well into 96-well plates. On the second day, the medium was changed to DMEM plus 10% FBS supplemented with 25HC (0 to 1,000 μM) and the cells were cultured for 24 to 72 h. CCK-8 (10 μL) was then added to each well, and the cell plates were incubated for 3 h at 37°C. The absorbance was detected at 450 nm with a microplate reader (model 680 microplate reader; Bio-Rad, USA).

### qRT-PCR analysis.

Total RNA was extracted from cells using the Omega total RNA kit, and cDNA was then synthesized using a TransStart one-step gDNA removal and cDNA synthesis supermix kit (TransGen Biotech) according to the manufacturer’s instruction. qRT-PCR was performed in triplicate using TransStart Tip Green qPCR supermix (TransGen Biotech) following the manufacturer’s protocols. The results were normalized against the level of GAPDH (glyceraldehyde-3-phosphate dehydrogenase) expression and quantified by the threshold cycle (2^−ΔΔ^*^CT^*) calculation method. The primers used for qRT-PCR were as follows: GAPDH-F (5′-ACATGGCCTCCAAGGAGTAAGA-3′) and GAPDH-R (5′-GATCGAGTTGGGGCTGTGACT-3′), PDCoV N-F (5′-CGCTTAACTCCGCCATCAA-3′) and PDCoV N-R (5′-TCTGGTGTAACGCAGCCAGTA-3′), ABCA1-F (5′-CTGCCTCCTCCACAGAGAAAAC-3′) and ABCA1-R (5′-CAGGGAAAACCCACCATACCT-3′), ACAT1-F (5′-CTGGGTGCAGGCTTACCTAT-3′) and ACAT1-R (5′-ACATGCTCTCCATTCCACCTG-3′), ACAT2-F (5′-AGCAGGTTGGTCACTGGAAG-3′) and ACAT2-R (5′-TCCTCCTTCAGTGTTGACCTTT-3′), SREBF2-F (5′-TTGTCGGGTGTCATGGGCG-3′) and SREBF2-R (5′-ATTGCAGCATCTCGTCGATGT-3′), and IFN-β-F (5′-TTCGAGGTCCCTGAGGAGATT-3′) and IFN-β-R (5′-TCCATCTGCCCATCAAGTTCC-3′).

### Western blotting.

The cells were lysed in radioimmunoprecipitation assay (RIPA) buffer supplemented with a protease and phosphatase inhibitor cocktail (HY-K0010 and HY-K0022; MedChemExpress) on ice for 15 min. Protein samples were separated by SDS-PAGE and then transferred to polyvinylidene fluoride membranes (Millipore, Bedford, MA, USA). After being blocked in 5% skim milk (A600669; Sangon Biotech) at room temperature for 2 h, the membrane was incubated with the primary antibody overnight at 4°C and then with the appropriate HRP-conjugated secondary antibody for 1 h at room temperature. The target protein bands were detected with the ECL enhanced chemiluminescence detection system (Tanon 6200 chemiluminescence imaging workstation; Tanon Science & Technology Co., Ltd., Shanghai, China). The bands were then quantified by densitometry using ImageJ software.

### Immunofluorescence analysis.

Cells cultivated on climbing sheets were fixed with 4% paraformaldehyde (PFA) in phosphate-buffered saline (PBS) for 30 min at room temperature. The cells were permeabilized in PBS plus 1% Triton X-100 (Sigma-Aldrich, St. Louis, MO, USA) for 15 min and blocked with 2% bovine serum albumin for 1 h at room temperature. After the cells were washed three times with PBS, the primary antibodies were incubated with the cells at 4°C overnight. The cells were then incubated with the appropriate Alexa Fluor-conjugated secondary antibodies for 1 h at room temperature. DAPI (Solarbio Science & Technology) was used to stain cell nuclei. The images were captured under a confocal laser scanning microscope (Nikon A1).

### Virus titration.

LLC-PK1 cells were grown in 12-well plates and then inoculated with PDCoV at an MOI of 0.1 or 1 for different time points. Then cell supernatants were collected and the cells were harvested for RNA extraction and immunofluorescence. LLC-PK1 cells were seeded into 96-cell plates and grown to 100% confluence for 24 h. Then, viral samples were serially diluted (10-fold) in DMEM supplemented with 0.4% trypsin and added to the LLC-PK1 cells in eight replicates per dilution. Cytopathic effect (CPE) was measured after 72 h of incubation, and viral titers were calculated as 50% tissue culture infective dose (TCID_50_) using the Reed-Muench method.

### Time-of-addition analysis.

After the inoculation of PDCoV (MOI = 0.1) as described above, 50 μM 25HC was added at different time intervals of 2 to 4, 4 to 6, 6 to 8, 8 to 10, and 10 to 12 h postinfection (hpi). The cell lysates were harvested for qRT-PCR analysis.

### BODIPY staining.

For BODIPY staining, the cells were fixed in 4% PFA for 30 min and then incubated with 2 μM BODIPY 493/503 (493-nm excitation/503-nm emission) at 37°C in the dark for 15 min. The digital images were obtained with a Nikon A1 confocal laser scanning microscope. The diameter and number of lipid droplets were determined with ImageJ software.

### Identification of COVID-19/SARS/MERS-associated genes.

Genes related to COVID-19, SARS, and MERS were collected from the GeneCards database, OMIM database, and NCBI gene function module. After that, these genes were compared to obtain the overlapping genes associated with COVID-19, SARS, and MERS and plotted as a Venn diagram.

### Enrichment analyses and network visualization.

Intersecting gene analysis and graphical plots were carried out under the R statistical environment R Studio (49). R programming language packages, including “ClusterProfiler,” “AnnotationDbi,” “org.Hs.eg.Db,” and “ggplot2,” were used for enrichment analyses and visualization of Gene Ontology (GO) biological process (BP), molecular function (MF), cellular component (CC), and KEGG pathways of the intersecting genes (50–54). GO information was obtained using “org.Hs.eg.Db.” A *P* value cutoff of 0.05 and *q* value cutoff of 0.05 were set for enrichment, and output was used for plotting the bubble chart, bar chart, and network plot.

### Statistical analysis.

GraphPad Prism 9 was used to perform statistical analysis. Data were obtained from at least three independent experiments for quantitative analyses and expressed as means ± standard deviation (SD). All statistical analyses were performed with the two-tailed Student's *t* test or one-way analysis of variance (ANOVA). *P* values of <0.05 were considered statistically significant.
